# The complete mitochondrial genome of *Limassolla lingchuanensis* (Hemiptera: Cicadellidae: Typhlocybinae)

**DOI:** 10.1080/23802359.2019.1698354

**Published:** 2019-12-12

**Authors:** Xiaowei Yuan, Kangning Xiong, Can Li, Yuehua Song

**Affiliations:** a School of Karst Science, Guizhou Normal University, Guiyang, Guizhou, China;; b State Engineering Technology Institute for Karst Desertification Control, Guiyang, Guizhou, China;; c Guizhou Provincial Key Laboratory for Rare Animal and Economic Insect of the Mountainous Region, Guiyang University, Guiyang, Guizhou, China

**Keywords:** *Limassolla lingchuanensis*, leafhopper, mitochondrial genome

## Abstract

The complete mitochondrial genome of the leafhopper *Limassolla lingchuanensis* was determined. It is 15,716 bp in length and consists of 13 protein-coding genes (PCGs), 22 transfer RNA (tRNA) genes, 2 rRNA genes, and a putative control region. ATN and TTG were initiation codons, and TAA, TAG, and T were termination codons. The phylogenetic relationships based on the neighbor-joining method were revealed using 13 PCGs with 10 leafhopper species of family Cicadellidae, which agree with the conventional taxonomy.

The leafhopper species *Limassolla lingchuanensis* (Chou and Zhang 1985) belongs to the subfamily Typhlocybinae (Hemiptera: Cicadellidae), the genus *Limassolla* includes 44 known species distributed across the world (Md et al. 2019). Many leafhopper species in this genus are all herbivorous insects with a broad diet and can feed on a variety of plants (Zhang et al. 2011). In this study, all examined samples were collected from Luodian in Guizhou Province of China (N25°56′, E106°86′). The whole body specimen was preserved in ethanol and stored in the insect specimen room of Guizhou Normal University with an accession number GZNU-ELS-2019001.

The overall base composition is 43.18% A, 35.64% T, 11.62% C, and 9.56% G, with A + T the complete mitogenome of *L. lingchuanensis* (GenBank accession number MN605256) is a typical closed-circular molecule of 15,716 bp in length, containing 13 protein-coding genes (PCGs), 22 transfer RNA genes (tRNAs), 2 rRNA genes (*rrnL* and *rrnS*), and a putative control region. The orientation and gene order of *L. lingchuanensis* are identical with other leafhoppers’ mitogenomes (Xing and Wang 2019; Yang et al. 2019). Thirteen genes were coded on the minor strand (J-strand), whereas the others were oriented on the major strand (N-strand). Gene overlaps in the *L. lingchuanensis* mitogenome were found at 16 gene junctions and involved a total of 45 bp with length varying from 1 to 8 bp. The longest overlap was situated between *trnC* and *trnW*. There are 11 intergenic spacer sequences in a total of 47 bp and the largest overlap is 26 bp long intergenic spacer, which is located between *nad5* and *trnH*.

The overall nucleotide composition was A (43.18%), T (35.64%), C (11.62%), and G (9.56%), The AT-skew and GC-skew of this genome were 0.0957 and −0.0972, respectively. The A + T content of the 13 PCGs ranged from 70.37% (*cox1*) to 83.01% (*atp8*). The length of 22 tRNAs ranged from 53 bp (*trnH*) to 71 bp (*trnK*), and A + T content varied from 73.02% (*trnS1*) to 89.06% (*trnG*). The *rrnL* and *rrnS* were spaced by trnV, with a length of 1168 and 743 bp, respectively. And all of them were encoded on the J-strand. The control region was 1416 bp length and 91.38% A + T content.

In the mitogenome of *L. lingchuanensis*, the total length of 13 PCGs is 10,787 bp, which accounts for 75.26% of the total genome. The *nad4* initiated with A as the start codon, *atp8* began with TTG, *nad3*, *nad5*, and *nad6* started with ATA, *cox2*, *nad1*, and *nad4l* started with ATT, and remaining six PCGs started with ATG. Nine PCGs including *atp6*, *atp8*, *cox3*, *nad1*, *nad3*, *nad4l*, *nad5*, *nad6*, and *cob* are terminated with TAA as stop codon, *cox1* and *nad2* end with TAG, *cox2* and *nad4* end with a single T residue. We analyzed the amino acid sequences of 13 PCGs with neighbor-joining method to reveal the phylogenetic relationship of *L. lingchuanensis* with other leafhoppers in family Cicadellidae. The result shows that *L. lingchuanensis* forms a clade with *Empoasca vitis* (Figure 1), both of them belongs to the subfamily Typhlocybinae. It is consistent with the traditional classification.

**Figure 1.**
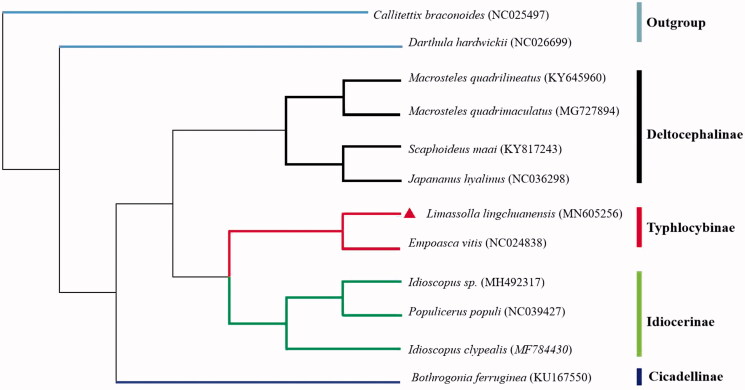
The neighbor-joining phylogenetic tree of *L. lingchuanensis* and 9 other leafhoppers in inner group. *Callitrttix braconoides* and *Darthula hardwickii* were used as an outgroup. GenBank accession numbers of each species are listed in the tree.
